# Chemoprevention of age-related macular regeneration (AMD) with rapamycin

**DOI:** 10.18632/aging.100469

**Published:** 2012-07-14

**Authors:** X. F. Steven Zheng

**Affiliations:** Department of Pharmacology and the Cancer Institute of New Jersey, Robert Wood Johnson Medical School (RWJMS), Piscataway, NJ 08854 USA

Age-related macular degeneration (AMD) is a leading cause of irreversible blindness among people of 50 year or older. During AMD, vascular endothelial growth factor (VEGF) may become elevated, causing choroidal neovascularization (CNV) and vascular permeability and fragility. Choroidal neovascularization can break in Bruch's membrane, resulting in subretinal hemorrhage, fluid exudation, detachment of the retinal pigment epithelium from the choroid, and formation of fibrotic scars [1]. Patients with neovascular age-related macular degeneration could suffer sudden visual loss due to subretinal hemorrhage or fluid accumulation secondary to choroidal neovascularization. Although neovascular AMD only accounts for less than 15% of the overall age-related macular degeneration, it is responsible for over 80 percent of the severe vision loss cases. Current therapeutics for neovascular AMD are two closely related antibody drugs, ranibizumab (trade name Lucentis) and bevacizumab (trade name Avastin), both of which bind to and neutralize VEGF [2,3]. Bevacizumab is a humanized anti-VEGF monoclonal antibody while ranibizumab is a humanized monoclonal antibody fragment that is made based on the same murine monoclonal antibody as bevacizumab. Ranibizumab is an FDA-approved drug but bevacizumab is used off-label by ophthalmologists. Both agents are used for intravitreal injections, which is cumbersome for patients and is not suitable for preventive treatment. Therefore, new drugs are needed to prevent and treat this type of disease.

It was reported in 2004 that rapamycin (trade name sirolimus) treatment significantly reduced the extent of neovascularization in both choroidal neovascularization and retinal neovascularization models induced in adult mice with laser photocoagulation and hyperoxia/hypoxia, respectively [4]. In an advance online publication this year in *American Journal of Pathology* [5], Kolosova *et al* presented exciting results that rapamycin could actually prevent AMD-like retinopathy in an aging rat model that more closely resembles human AMD pathology. They investigated the effect of rapamycin on spontaneous retinopathy in senescence- accelerated OXYS rats. OXYS rats were treated orally with either 0.1 or 0.5 mg/kg rapamycin, which was given together with food. Rapamycin was found in a dose-dependent manner to reduce the incidence and severity of retinopathy, and attenuated AMD disease progression. Some histological abnormalities associated with retinopathy were notably reduced. For examples, in retinal pigment epithelial cell layers, rapamycin decreased nuclei heterogeneity and normalized intervals between nuclei; in photoreceptor cells, rapamycin prevented nuclear and cellular pyknosis; significantly, rapamycin prevented destruction of ganglionar neurons in the retina. Rapamycin did not exert any adverse effects on the retina in control disease-free Wistar rats, suggesting that it is safe.

Rapamycin is a macrolide antibiotic and FDA-approved drug for organ transplantation, prevention of restenosis in cardiology and treatment of advanced renal cancer [6]. Chronic oral use of rapamycin is well tolerated in human patients, with mild side effects mainly being elevated triglycerides. These new results demonstrate a potential therapeutic utility of rapamycin for treatment and prevention of retinopathy in AMD and severe diabetes. Although rapamycin was previously reported to block VEGF expression [7], it did not significantly decrease VEGF levels in the murine AMD models [4,5], suggesting that rapamycin acts through a different mechanism. One possibility is that rapamycin inhibits VEGF stimulated intracellular signaling necessary for angiogenesis, which requires mTOR, the mechanistic target of rapamycin [8]. Evidence also accumulates that mTOR is a key aging factor. Genetic down-regulation of mTOR or rapamycin treatment in various model organisms ranging from the yeast *S. cerevisiae*, the worm *C. elegans*, the fruit fly *D. melanogaster*, and genetically heterogeneous mice, were found to significantly extend the lifespan of these organisms [6]. Moreover, rapamycin is known to reduce many age-related diseases such as AMD and Parkinson's disease [6]. Conceivably the AMD-preventive effect could be attributed to the overall anti-aging activity of rapamycin. Because diseases such as AMD are difficult to cure once they have developed, early chemoprevention is likely the best option. The findings that rapamycin prevents the progression of AMD in a more physiologically model provide a new hope for the high risk AMD population.
